# Systematic Review of the Efficacy of Orbital Atherectomy in Improving the Outcome of Percutaneous Corornary Intervention in People With Diabetes

**DOI:** 10.7759/cureus.50153

**Published:** 2023-12-08

**Authors:** Chioma G Muoghalu, Cosmas C Ofoegbu, Ndianabasi E Ekong, Danvictor A Ebirim, Sandra T Alex-Ojei, Foziyah Alqahtani

**Affiliations:** 1 Medicine, University of Galway, Galway, IRL; 2 Health Sciences, Central Washington College, Enugu, NGA; 3 Community and Family Medicine, Allith General Hospital, Allith, SAU; 4 Medical Center, Akwa Ibom State College of Education, Afaha Nsit, NGA; 5 Department of Medicine, Federal University Teaching Hospital, Owerri, NGA; 6 Department of Medicine, University of Port Harcourt Teaching Hospital, Port Harcourt, NGA; 7 Department of Cardiac Technology, Imam Abdurahman Bin Faisal University, Dammam, SAU

**Keywords:** orbital atherectomy devices, orbital atherectomy, pci, percutaneous coronary intervention, diabetic patients, diabetes mellitus, dm

## Abstract

The optimal approach to deal with severe coronary artery calcification (CAC) in people with diabetes remains ill-defined. People with diabetes have a significant risk of developing severe vessel calcification and coronary artery disease (CAD). CAD is the leading cause of death in people with diabetes. Individuals with diabetes mainly present with severe multivessel stenosis, diffuse coronary calcification, and severe atherosclerosis, which are poor prognostic factors of revascularization procedures. Studies have shown that the revascularization of arteries in people with diabetes often results in worse outcomes than in people without diabetes. Coronary artery bypass grafting (CABG) has been recommended as the standard of care for people with DM and complex anatomic diseases, including left main CAD. However, percutaneous coronary intervention (PCI) is more acceptable to patients in clinical practice because of decreased trauma and rapid recovery. Severe CAC has traditionally been challenging for PCI and a frequent indication for surgical revascularization.

This study aims to determine the effectiveness of orbital atherectomy (OA) in improving PCI outcomes in patients with diabetes and identify possible adverse effects that preclude its use.

The study is reported according to PRISMA and analyzed according to Cochrane guidelines on synthesis without meta-analysis. A comprehensive literature search of EMBASE, Scopus, Web of Science, Cochrane Library, CINAHL, and MEDLINE was conducted for studies that utilized OA before PCI in people with diabetes. A reference list of the eligible articles was also screened. A narrative synthesis was done by representing the data on the effect direction plot, followed by vote counting.

Eighteen studies were included in the analysis. Success rate/successful stent delivery was >90%, while freedom from angiographic complication and major adverse cardiovascular and cerebrovascular events (MACCE) were both >80% on the effect direction plot for people with diabetes and those without diabetes.

People with diabetes had low event rates similar to those without diabetes. OA appears to be a viable treatment approach for people with diabetes. However, RCTs with a longer duration of follow-up are required to establish the appropriate treatment strategy for severe CAC in people with diabetes.

## Introduction and background

One of the compelling hurdles of percutaneous coronary intervention (PCI) is vessel calcification [1]. In people with diabetes mellitus (DM), the optimal revascularization strategy for complex artery disease remains an essential issue for cardiovascular experts [1]. A pointer to advanced artery disease is the presence of calcification; the extent of calcification correlates strongly with the degree of atherosclerosis and cardiac events [2-5]. Worldwide, the incidence of DM has been increasing rapidly [6,7]. The total number of people with DM is expected to increase to 600 million by 2035 [8]. DM is a critical risk factor for severe vessel calcification and coronary artery disease (CAD) [9]. People with diabetes mainly present with severe multivessel stenosis, diffuse coronary calcification, and severe atherosclerosis, which are poor prognostic factors of revascularization procedures [10-12]. Studies have shown that the revascularization of arteries in people with diabetes often results in worse outcomes than in people without diabetes [13-15].

Coronary artery bypass grafting (CABG) has been recommended as the standard of care for people with DM and complex anatomic diseases, including left main CAD [16]. However, PCI is more acceptable to patients in clinical practice because of decreased trauma and rapid recovery [1]. Although PCI is more prone to a greater rate of adverse events after revascularization [1], some studies have revealed no difference in mortality between PCI and CABG [17]. On the other hand, CABG is more invasive than PCI and has a higher risk of excess stroke [18-20], making it difficult to assess the two strategies. PCI of severely calcified vessels is associated with poor outcomes, including angiographic complications, incomplete stent expansion, and restenosis [9,21-23]. However, with the use of drug-eluting stents (DESs) and atherectomy devices, the rate of restenosis and repeat revascularization after percutaneous coronary intervention (PCI) has markedly reduced [24,25]. Therefore, PCI is regarded as an alternative to CABG as its outcome has improved considerably; it's less invasive and favored more by patients.

Severe coronary artery calcification (CAC) has traditionally been challenging for PCI and a frequent indication for surgical revascularization [1]. Since the introduction of plain balloon angioplasty 45 years ago, the interventional cardiology community has introduced several other devices (cutting balloon, scoring balloon, and atherectomy devices) to overcome the challenges associated with calcification [26]. However, adverse effects and sub-optimal results limited the use of these devices [26]. The proposed optimal approach for managing calcified lesions is a non-atherectomy technique for mild lesions and an atherectomy technique for heavily calcified lesions [26].

Atherectomy devices were invented to enable drilling, grinding, or sanding of atheroma, calcium, and excess cellular material from the location of coronary occlusion or stenosis [27]. Mechanical and laser-based strategies are utilized [27]. Historically, atherectomy devices have been used during revascularization procedures for calcified lesions [28]. Proactive preparation of calcified vessels with atherectomy devices facilitates successful stent delivery, implantation, and optimal stent expansion and improves outcomes [28]. Unfortunately, evidence indicates that atherectomy is utilized in less than 5% of PCI patients, even though studies have shown that the prevalence of coronary artery calcification in PCI patients is 32% [29,30] and up to 73% when intravascular ultrasound is used to assess calcification [31]. Thus, most patients with CAC are still treated with only drug-eluting stents (DES), leading to poor PCI outcomes. The commercially available coronary atherectomy devices include orbital atherectomy (OA), rotational atherectomy (RA), and laser atherectomy (LA) [28]. RA and LA have been used for decades; however, OA is the only atherectomy device explicitly indicated by the US FDA to treat severely calcified coronary lesions and improve stent delivery [28,32]. RA does not reduce recurrent in-stent restenosis after angioplasty [33]. Additionally, OA is associated with fewer post-procedural complications with decreased fluoroscopy time than RA [32]. OA reduces vessel calcium and facilitates stent delivery leading to increased procedural success, favorable long-term outcomes, and reduced revascularization rates [34].

## Review

Aims and objectives

Some studies have shown that OA effectively improves the outcome of revascularization procedures in people with diabetes. However, the efficacy of OA in people living with DM has not been reviewed systematically. Therefore, this study aims to summarize the existing data to evaluate the effectiveness of OA in improving PCI outcomes in people living with DM. The study reviewed available evidence on the efficacy of OA in improving PCI outcomes, determined the effectiveness of OA in improving PCI in people with DM, identified possible adverse effect that precludes the use of OA, assessed the quality of evidence on OA, and measured the outcomes of PCI following OA in DM patients.

Device description

The coronary orbital atherectomy device manufactured by Cardiovascular Systems, Inc. is a percutaneous device that enhances stent delivery in heavily calcified coronary lesions [35]. It uses a high-speed orbiting diamond-coated crown that sands the hard calcified plaque away. At the same time, the soft vessels flex away from the crown, leading to maximal vessel compliance and minimal tissue injury [35]. 

Methodology

The protocol and registration number of this study is CRD42022308685. The study is reported following the 2020 Preferred Reporting Items for Systematic Reviews and Meta-Analysis Extension (PRISMA) statement [36], analyzed according to Cochrane guidelines on synthesis without meta-analysis (SWIM) [37] and critically appraised using the CASP cohort study checklist [38]. All available data are quantitative studies.

Inclusion Criteria 

Type of study: Eligible studies assessed the efficacy of OA in patients living with DM. No restriction was placed on the date of publication, the study design, follow-up duration, and sample size owing to the relative novelty of the approach. Studies that were not designed for people with diabetes a priori but had diabetes as a comorbidity were also added for the same reason.

Population: Studies focused on all patients with diabetes or who have diabetes as a comorbidity were included.

Exposure: Eligible studies evaluated the efficacy of orbital atherectomy prior to PCI.

Outcome: Eligible studies reported at least one component of angiographic complication and major adverse cardiovascular and cerebrovascular event (MACCE), which is defined as all-cause death, myocardial infarction (MI), target vessel revascularization (TVR), and cerebrovascular event. MI is defined as recurrent symptoms with new-onset ST-segment elevation or re-elevation of cardiac markers to at least twice the upper limit of normal. TVR is defined as repeat revascularization of any segment of the target vessel. The primary efficacy endpoint is successful stent delivery without severe angiographic complications. Angiographic complications included dissections, perforation, persistent slow flow, persistent no-reflow, embolization, and abrupt closure. Outcomes reported during the hospital stay or within 30 days of the index procedure were considered short-term outcomes while events occurring after 30 days of the index procedure are defined as long-term outcomes.

Exclusion Criteria

Overlapping/repetitive studies were excluded. Studies focused on PCI done with other atherectomy devices were eliminated. Non-English studies were not included.

Search Strategy

A comprehensive literature search was conducted on EMBASE, Scopus, Web of Science, Cochrane Library, CINAHL, and MEDLINE databases using key search terms defined by the PEO (population, exposure, and outcome) framework. Boolean operators ("AND" "OR") were used as a search strategy. The search string included the following keywords: (DM OR diabetes mellitus OR diabetic patients) AND (percutaneous coronary intervention OR PCI) AND (orbital atherectomy OR orbital atherectomy devices).

Study Selection

The study selection was made in three stages of screening, namely title screening, abstract, and full-text. To ensure all relevant articles are included, study selection was conducted by two independent investigators. Inconsistency in opinion was resolved by discussion with other reviewers.

Data Extraction

Titles and abstracts of all relevant studies were merged into the Mendeley software, and all duplicates were excluded. Data extraction was done by two independent reviewers. The extracted data was crosschecked by other reviewers. The data included author, publication date, design of studies, number of patients, setting, follow-up duration, and outcome. See Tables 1-4 for study characteristics/patient demographics, lesion characteristics, CASP cohort checklist/quality assessment, and outcome measures.

**Table 1:** Study Characteristics EF: ejection fraction, NR: not recorded, HLD: hyperlipidemia, DM: diabetes mellitus, NDM: non-diabetes mellitus, HF: heart failure, CKD: chronic kidney disease, HTN: hypertension, BMI: body mass index, NRCT- Non-randomized controlled trial, MACCE: major adverse cardiovascular and cerebrovascular events

Author/ Year/ Country	Sample Size	Study Design, Setting	Mean age (years)	Baseline Patient Characteristics	Follow up	Outcome
Chambers et al., 2022 [39], USA	56 (DM=31)	Retrospective, Multicentre	72	Complex and heavily calcified ostial lesion with a high prevalence of DM (55%)	30 days, one year, two years	Low rate of angiographic complications and MACCE
Rao et al., 2021 [40], USA	221 (DM=96)	Retrospective, Multicentre	DM- 71.8 NDM- 72.8	DM – younger & lower EF, Higher prevalence of CKD, HTN, HLD, HF in DM	In hospital 30 days	High rate of successful stent delivery in both DM & non-DM. High rate of procedural success in both DM & non-DM. Low rate of angiographic complications & MACCE in both DM & non-DM.
Beohar et al., 2021 [41], USA	609 (DM=264)	Retrospective, Single-centre	74	Compared Hispanic and non-Hispanic patients. Hispanics had a higher prevalence of DM and complex lesions.	In-hospital	Both groups had a low rate of angiographic complications and MACCE
Sturm et al., 2020 [42], USA	151 (DM=87; 57%)	Retrospective, Single-centre	70.6	Comorbidities include – DM, HTN, HLD, HF, and MI. Prior CABG – 23.2% Prior PCI – 51% EF – 46.2 (mean)	30 days, Six months, One year	High rate of successful stent delivery (100%) & procedural success. Low rates of successful complications & MACCE in both DM and non-DM.
Schlofmitz et al., 2019 [43], USA	309 (DM=121)	Retrospective, Single-centre	73	Heavily calcified lesions with a high number of DM (39.4%)	In-hospital 30 days	Low rate of adverse events
Lee et al., 2018 [44], USA	443 (DM=160)	Prospective, Multicentre	DM-70.3 NDM-72	DM-Younger with lower EF & higher BMI. Higher prevalence of CKD, HTN, HLD, Stroke, MI, prior CAGB, & PCI in DM...	30 days, One year	Procedural success was high & same in both groups Low rates of angiographic complications & MACCE in both groups.
Lee et al., 2018 [45], USA	458 (DM=183)	Retrospective, Multicentre	75	Heavily calcified lesions with a high prevalence of DM (39.9%)	30 days	High procedural success rate and low rate of adverse events
Lee et al., 2018 [46], USA	458 (DM=192)	Retrospective, Multicentre	74	Heavily calcified lesions with a high prevalence of DM (42.1%)	One year	High procedural success rate and low rate of adverse events
Lee et al., 2018 [47], USA	64 (DM=28)	Retrospective, Multicentre	76.2	Complex lesions and comorbidities with increased DM patients (45.2)	One year	High procedural success rate and low rate of adverse events
Rupak et al., 2018 [48], USA	40 (DM=22)	Retrospective Single-centre	68.5	Heavily calcified lesions with a high prevalence of DM (55%)	6.5 months (197.5 days)	High procedural success rate and low rate of adverse events
Whitbeck et al., 2018 [49], USA	70 (DM=28)	Retrospective Single-centre	DM- 74.4 NDM- 70.6	DM-Older with higher EF. Higher prevalence of CKD & HTN in DM. Other comorbidities were higher in non-DM	In-hospital	High rate of successful stent delivered and a procedural low rate of angiographic complications & acute MACCE in both groups.
Lee et al., 2017 [50], USA	456 (DM=192)	Retrospective, Multicentre	NR	Compared patients with and without CKD: patients with CKD had higher DM and HTN.	30 days	Low rate of angiographic complications & MACCE among all groups
Lee et al., 2017 [13], USA	443 (DM=160)	Prospective, Multicentre	DM-70.3 NDM-72	DM-Younger with lower EF & higher BMI. Higher prevalence of CKD, HTN, HLD, stroke, MI, prior CAGB, & PCI in the DM group.	Three years	Procedural success was high & same in both groups. Low rates of angiographic complications & MACCE in both groups.
Genereux et al., 2016 [51], USA	443 (DM=160)	Prospective, Multicentre	DM-70.3 NDM-72	DM-Younger with lower EF & higher BMI. Higher prevalence of CKD, HTN, HLD, stroke, MI, prior CAGB, & PCI in DM.	Two years	Procedural success was high & same in both groups. Low rates of angiographic complications & MACCE in both groups.
Lee et al., 2016 [9], USA	458 (DM=193)	Retrospective, Multicentre	DM-71.9 NDM-75.1	DM-Younger with lower EF, higher prevalence of CKD, HTN, HLD, MI in DM group.	30 days	Low rate of Angiographic complications & MACCE among all groups
Bhatt et al., 2015 [52], India	33 (DM=10)	Prospective, Single-centre NRCT	54	Heavily calcified lesions with a high number of DM (30.3%)	Five years	High procedural success rate and low rate of adverse events
Bhatt et al., 2014 [53], India	33 (DM=10)	Prospective, Single-centre NRCT	54	Heavily calcified lesions with a high number of DM (30.3%)	Three years	High procedural success rate and low rate of adverse events
Keyur et al., 2013 [54], India	50 (DM=20)	Prospective, Multicentre NRCT	57.4	Heavily calcified lesions with a high prevalence of DM (40%)	In-hospital 30 days, six months	High procedural success rate and low rate of adverse events

**Table 2:** Lesion characteristics NR: not recorded, NDM: no diabetes mellitus, LM: left main vessel, CTO: chronic total occlusion, ISR: In-stent restenosis.

Study	Chambers et al., 2022 [39]	Rao et al., 2021 [40]		Beohar et al., 2021 [41]	Sturm et al., 2020 [42]	Shlofmitz et al., 2019 [43]	Whitbeck et al., 2018 [49]		Lee et al., 2018 [44]		Lee et al., 2018 [47]	Lee et al., 2018 [45]	Lee et al., 2018 [46]	Lee et al., 2017 [50]	Lee et al., 2017 [13]		Lee et al., 2016 [9]		Genreux et al., 2016 [51]		Bhatt et al., 2015 [52]	Bhatt et al., 2014 [53]	Keyur et al., 2013 [54]	Rupak et al., 2018 [48]
Population	ALL	DM	NDM	ALL	ALL	ALL	DM	NDM	DM	NDM	ALL	ALL	ALL	ALL	DM	NDM	DM	NDM	DM	NDM	ALL	ALL	ALL	ALL
Multi Vessel PCI (%)	23.2	7.3	4.0	NR	9.3	NR	NR	NR	NR	NR	NR	NR	NR	NR	NR	NR	14.5	13.2	NR	NR	NR	NR	NR	7.5
OSTIAL (%)	100	1.0	1.6	NR	15.9	NR	NR	NR	NR	NR	NR	NR	NR	NR	NR	NR	NR	NR	NR	NR	NR	NR	NR	NR
LM (%)	12.5	1.0	1.6	4.3	NR	2.1	7.1	7.1	3.8	1.4	100	3.9	NR	NR	3.8	1.4	4.1	3.8	3.8	1.4	NR	NR	NR	15
TORTOUS (%)	1.8	2.1	1.6	NR	NR	NR	NR	NR	NR	NR	NR	NR	NR	NR	NR	NR	NR	NR	NR	NR	NR	NR	NR	NR
ISR (%)	NR	1.0	1.8	NR	NR	NR	NR	NR	NR	NR	NR	NR	NR	NR	NR	NR	NR	NR	NR	NR	NR	NR	NR	NR
Bifurcation (%)	26.9	NR	NR	NR	15.9	NR	35.7	31.0	35.7	31.0	71	NR	NR	NR	35.7	31.0	NR	NR	35.7	31.0	NR	NR	NR	NR
CTO (%)	3.6	NR	NR	NR	2.0	NR	0	7.1	0	7.1	NR	NR	NR	NR	0	7.1	NR	NR	0	7.1	NR	NR	NR	NR
CLASS C LESION (%)	NR	NR	NR	54	64.9	NR	NR	NR	71.4	69	NR	NR	NR	NR	71.4	69	NR	NR	71.4	69	NR	NR	NR	NR
Stent Diameter (Mean mm)	NR	3.1	3.2	NR	NR	NR	NR	NR	NR	NR	NR	NR	NR	NR	NR	NR	3.1	3.1	NR	NR	NR	NR	NR	NR
Vessel Diameter (Mean mm)	NR	NR	NR	NR	2.9	NR	NR	NR	3.1	3.1	NR	3	NR	NR	3.1	3.1	NR	NR	3.1	3.1	NR	NR	NR	NR
Stent length (Mean mm)	NR	26.4	24.4	NR	NR	40.4	NR	NR	NR	NR	NR	40	NR	NR	NR	NR	44.2	43.8	NR	NR	22	22	NR	NR
Lesion length (Mean mm)	29.9	NR	NR	22.6	28.4	NR	NR	NR	19.3	18.7	NR	NR	NR	NR	19.3	18.7	NR	NR	19.3	18.7	15.9	15.9	13.4	32.5
Severe Calcification (%)	100	93.8	96	NR	99.3	NR	NR	NR	90.6	92.6	NR	NR	NR	NR	90.6	92.6	NR	NR	90.6	92.6	NR	NR	NR	NR

**Table 3:** CASP-COHORT study checklist ✓ = Yes X = No ? = Can't tell

Criteria	Chambers et al., 2022 [39]	Rao et al., 2021 [40]	Beohar et al., 2021 [41]	Sturm et al., 2020 [42]	Shlofmitz et al., 2019 [43]	Whitbeck et al., 2018 [49]	Lee et al., 2018 [44]	Lee et al., 2018 [45]	Lee et al., 2018 [46]	Lee et al., 2018 [47]	Rupak et al., 2018 [48]	Lee et al., 2017 [13]	Lee et al., 2017 [50]	Lee et al., 2016 [9]	Genereux et al., 2016 [51]	Bhatt et al., 2015 [52]	Bhatt et al., 2014 [53]	Keyur et al., 2013 [54]
Focused	✓	✓	✓	✓	✓	✓	✓	✓	✓	✓	✓	✓	✓	✓	✓	✓	✓	✓
Acceptable recruitment	✓	✓	✓	✓	✓	✓	✓	✓	✓	✓	✓	✓	✓	✓	✓	✓	✓	✓
Accurate measurement of exposure	✓	✓	✓	✓	✓	✓	✓	✓	✓	✓	✓	✓	✓	✓	✓	✓	✓	✓
Identification of confounding factors	✓	✓	✓	✓	✓	✓	X	✓	✓	✓	✓	?	✓	✓	✓	✓	✓	✓
Consideration of confounding factors	✓	X	✓	✓	✓	✓	X	✓	✓	✓	✓	?	✓	✓	✓	✓	✓	✓
Adequate duration of follow-up	✓	X	X	✓	X	X	✓	✓	X	✓	✓	X	✓	X	✓	✓	✓	✓
Result	✓	✓	✓	✓	✓	✓	✓	✓	✓	✓	✓	✓	✓	✓	✓	✓	✓	✓
Precise result	✓	✓	✓	✓	✓	✓	✓	✓	✓	✓	✓	✓	✓	✓	✓	✓	✓	✓
Reliable result	✓	✓	✓	✓	✓	✓	✓	✓	✓	✓	✓	✓	✓	✓	✓	✓	✓	✓
Generalizable	✓	?	✓	✓	✓	?	?	✓	✓	✓	✓	✓	✓	?	✓	?	?	✓
Correspond with the available evidence.	✓	✓	✓	✓	✓	✓	✓	✓	✓	✓	✓	✓	✓	✓	✓	✓	✓	✓
Practical implication	✓	✓	✓	✓	✓	✓	✓	✓	✓	✓	✓	✓	✓	✓	✓	✓	✓	✓

**Table 4:** Outcome NDM: no diabetes, TVR: target vessel revascularization, CTO: chronic total occlusion, LM: left main, ↑: high rate of successful stent delivery without mentioning the actual value, IN-HOS: in hospital, MACCE: major adverse cardiovascular and cerebrovascular event, MACE: major adverse cardiac event, CABG: Coronary artery bypass grafting, MI: myocardial infarction

Study	Chambers et al., 2022 [39]	Rao et al., 2021 [40]		Beohar et al., 2021 [41]	Sturm et al., 2020 [42]	Shlofmitz et al., 2019 [43]	Whitbeck et al., 2018 [49]		Lee et al., 2018 [44]		Lee et al., 2018 [46]	Lee et al., 2018 [45]	Lee et al., 2018 [47]	Rupak et al., 2018 [48]	Lee et al., 2017 [13]		Lee et al., 2017 [50]	Lee et al., 2016 [9]		Genereux et al., 2016 [51]	Bhatt et al., 2015 [52]	Bhatt et al., 2014 [53]	Keyur et al., 2013 [54]
Outcome	2 Years	30 days		IN-HOS	1 Year	30 Days	ACUTE		1 Year		1 Year	30 Days	1 Year	6.5 Months	3 Years		30 Days	30 Days		2 Years	5 Years	3 Years	6 Months
Population	ALL	DM	NDM	ALL	ALL	ALL	DM	NDM	DM	NDM	ALL	ALL	ALL	ALL	ALL		ALL	DM	NDM	ALL	ALL	ALL	ALL
MACCE/MACE	12.2	2.1	0.8	1.5	12.3	1.06	0	2.4	17.1	16.7	11.3	NR	NR	10	23.5		2.1	1	3	19.4	21.1	18.2	8
Death (%)	10.3	NR	NR	0.7	8.1	0	0	0	3.9	2.9	8.1	1.4	4	7.5	12.4		1.3	0.5	1.9	7.5	12.1	9.1	2
MI (%)	0	2.1	0	0.8	2.9	0	0	2.4	9.4	11.3	8.1	1.2	1.8	0	11.2		1.09	0.5	1.5	NR	6.1	6.1	6
TVR (%)	2	1.00	0	NR	2.8	0	0	0	5.9	5.8	4.8	0	7.5	2.5	10.2		0	0	0	8.1	3	3	2
Stroke (%)	3.7	0	0	0.3	1.8	0	0	0	NR	NR	0	0.2	1.3	0	NR		0.2	0	0.4	NR	NR	NR	0
Stent Thrombosis (%)	0	2.1	0	NR	1.9	0	0	0	NR	NR	0	0.8	1.3	0	NR		0.8	0	1.5	NR	NR	NR	0
Angiographic Complication	ALL	DM	NDM	ALL	ALL	ALL	DM	NDM	DM	NDM	ALL	ALL	ALL	ALL	DM	NDM	ALL	DM	NDM	ALL	ALL	ALL	ALL
Emergency CABG (%)	NR	NR	NR	NR	NR	0	NR	NR	NR	NR	0	NR	NR	NR	NR	NR	0.65	0	0.4	NR	NR	NR	NR
Perforation (%)	5.4	0	0.8	0.8	1.36	0	0	2.4	NR	NR	0	0.6	0.7	2.5	NR	NR	0.65	0.5	0.8	NR	6.1	6.1	2
Dissection (%)	1.8	0	0	0.5	0.7	0	4.3	0	NR	NR	3.2	0.8	0.9	0	NR	NR	0.8	1.0	0.8	NR	3	3	1.2
Slow Flow (%)	0	0	0	0	NR	0	3.6	0	NR	NR	0	0	0	0	NR	NR	NR	NR	NR	NR	0	0	0
No Reflow (%)	0	0	0	0	0.7	0	0	0	NR	NR	0	0.6	0.7	0	NR	NR	0.65	1.0	0.4	NR	0	0	0
Residual Stenosis (%)	NR	2.2	2.2	0.8	0.9	NR	NR	NR	4.1	4.9	NR	NR	NR	NR	4.1	4.9	NR	NR	NR	NR	0.3	0.3	0.3
Successful Stent Delivery (%)	100	95.8	98.4	100	100	↑	96.4	100	97.7	97.7	100	↑	99.1	100	97.7	97.7	↑	↑	↑	97.7	100	100	94

Critical Appraisal/Quality Improvement

The quality of the articles was assessed using the CASP cohort study checklist [38] and graded as medium or high quality. See (table 3) and the result section for further details.

Data Synthesis/Analysis

**Figure 1:**
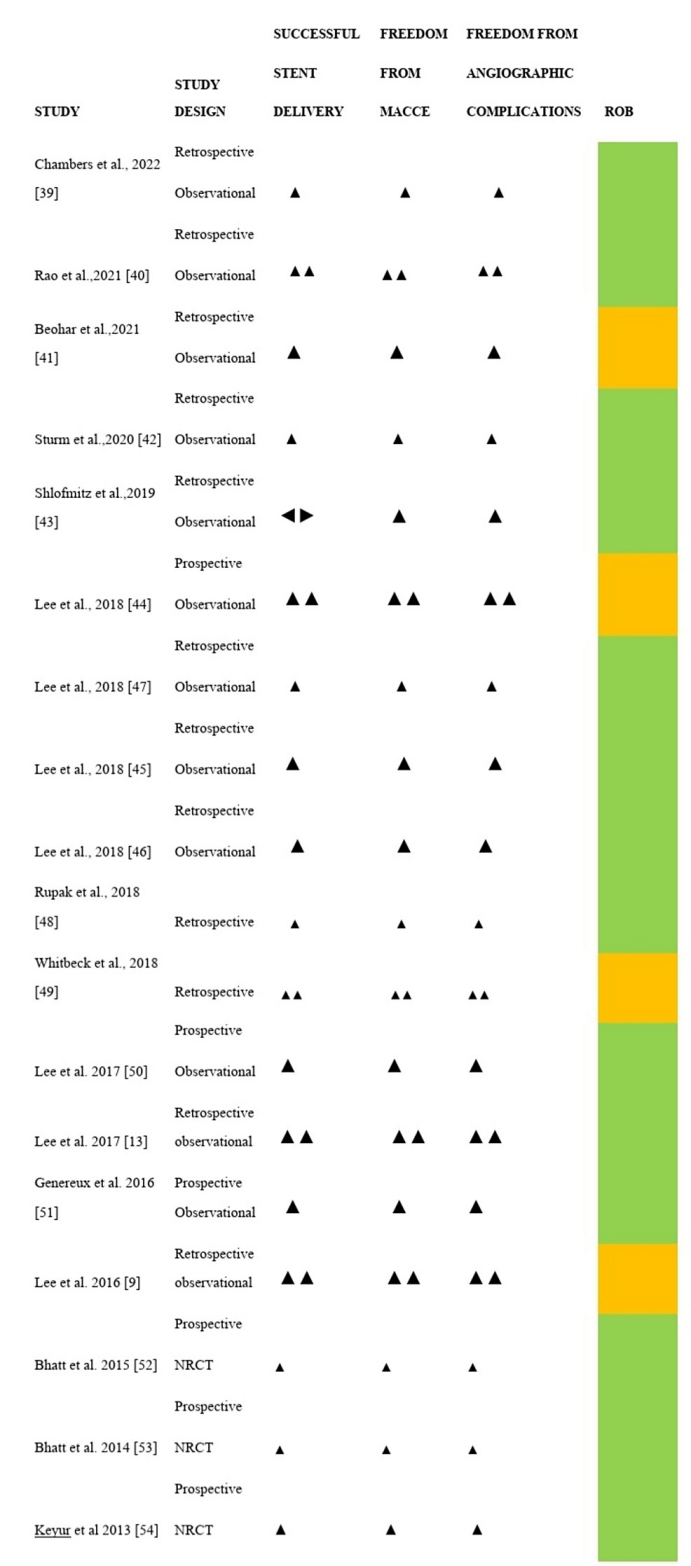
Effect direction plot [9,13,39-54] The upward arrow ▲ signifies positive health impact; the downward arrow ▼indicates negative health impact; the double arrow ▲▲ represents similar outcomes for studies that compared the outcome of DM and NDM patients; the bidirectional arrow ◄► suggests no recorded value by the study. Final sample size (individuals in the intervention group): the big arrow ▲ represents a sample size greater than 300, the medium arrow ▲ represents a sample size of 50-300, and the small arrow ▲ represents a sample size less than 50. The row color denotes the quality of the study: green denotes a low risk of bias, amber denotes some concerns regarding the quality, and red denotes a high risk of bias. NRCT- Non-randomized controlled trial.

Studies that met the eligibility criteria and passed the three-stage selection process were synthesized following the Cochrane guideline for SWIM [37]. An effect direction plot was used to present the result of the narrative data synthesis. An upward arrow signifies a positive health outcome (successful stent delivery, freedom from MACCE, and freedom from angiographic complications), while the bidirectional arrow suggests no recorded value by the study. The direction of the effect is reported if ≥70% of the outcome reports the same direction. The quality of the study is denoted by the row color. See Figure 1 and the result section for further details.

Result

**Figure 2:**
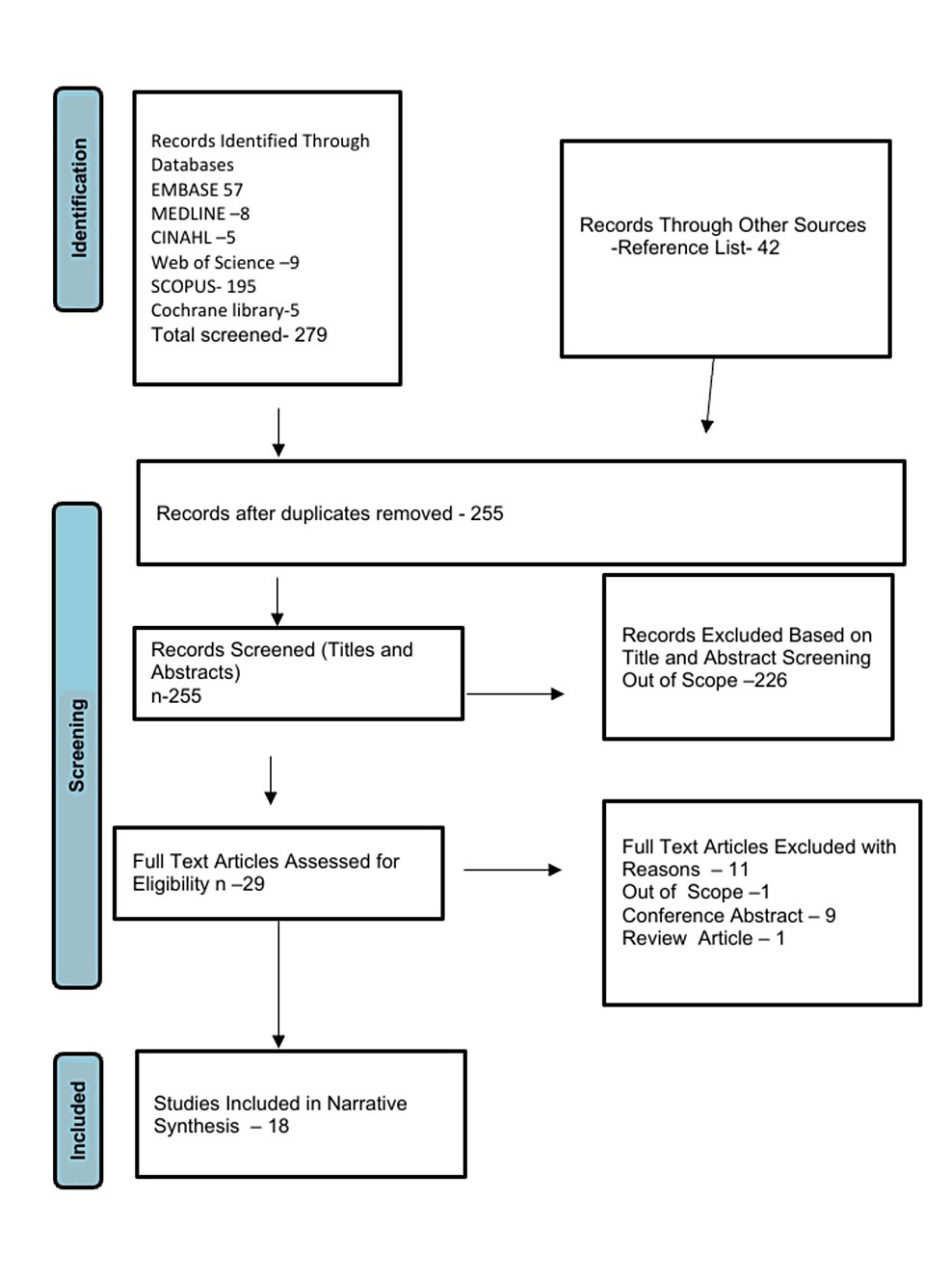
PRISMA diagram

The study selection process is shown in Figure 2.

Of the 321 references retrieved initially from the databases, only eighteen quantitative studies were selected for final analysis [9,13,39-54]. Twelve studies were retrospective [9,39-43,45-50] while six are prospective [13,44,51-54]. Six studies are non-randomized clinical trials (subanalysis and follow-ups of ORBIT 1 and 11 trials) [13,44,51-54]. Ten are multicenter studies [9,13,39,40,44-47,50,54] while the rest are single-centred [41-43,48,49,52]. The included studies were all conducted in the USA [9,13,39-51], except three which were conducted in India [52-54]. Six studies were designed to compare the outcome of OA in people with diabetes and those without DM [9,13,40,44,49,51], while the rest only included diabetes as a comorbidity.

All the studies were published between 2013 and 2022 and included 3843 patients, with 1617 individuals living with diabetes. The age range of people with diabetes was between 70.3 and 74.4 years, while that of people without diabetes was between 71.4 and 75.1 years). For the studies that included DM as a comorbidity, the age range was between 54 and 76 years. In the six studies designed for people with diabetes a priori, patients with diabetes were younger. They had lower ejection fractions except in the study by Whitbeck et al., where they are older with higher ejection fractions. There is a higher prevalence of hypertension (HTN), CKD, hyperlipidemia (HLD), stroke, MI, prior CABG, and PCI in the DM group in most of the studies (see Table 1). Eight studies had smaller cohorts [39-41,48,49,52-54], while the follow-up duration was noticeably short in seven studies [9,40,41,43,45,49,50].

The baseline study characteristics are illustrated in Table 1, while the baseline lesion/angiographic characteristics and outcome are outlined in Table 2. The studies did not report all the lesion characteristics that could affect the procedural outcome; nonetheless, the reported baseline characteristics are similar among people with and without DM.

Quality Assessment

Studies were assessed utilizing the CASP cohort study checklist (see Table 3). Although they were all observational studies, they were graded moderate to high because they took cognizance of confounding factors. Most cohorts presented with complex lesions and comorbidities with a favorable outcome.

Outcome Measure/Effect Direction Plot

The outcome measures of this study are portrayed in Table 4 and an effect direction plot (see Table 4 and Figure 1). The effect direction plot effectively visually represents the characteristics/outcome of studies in SWIM [55]. It also represents evidence of a positive, negative, or no change in effect. This study analyses the efficacy and safety of OA in improving PCI outcomes. Each outcome measure is represented on the plot without considering the p-value/statistical significance [55]. The efficacy endpoint was assessed by procedural success rate/stent delivery. On the other hand, the safety endpoint was recorded as freedom from (FF) MACE/MACCE and FF angiographic complications. FF MACE/angiographic complications were derived by subtracting the observed MACCE/angiographic complication from 100%.

Although only a few studies compared the outcome of people with DM to those without DM, all studies reported a high rate of successful stent delivery (only 14 studies recorded the actual value). Similarly, a low rate of angiographic complications and MACCE (high rate of FF angiographic complication and MACCE) were recorded by all studies, as shown below.

According to Boon and Thomson, 2021, when analyzing the result of the vote counting of an effect direction plot, ≥70% of the direction of an outcome measure has to report in a similar direction for the effect to be reported [55]. If it's less than 70%, the direction of the effect with the majority is reported; however, a conclusion of conflicting findings will be made [55]. The direction of effects in this plot are all upwards, which signifies a positive outcome. Based on this, our study concluded that OA is a safe and effective way of improving PCI outcomes in patients with diabetes.

Discussion

CAC remains an issue for interventional cardiologists. Over the past four decades, numerous devices have been invented to overcome the problem of CAC. Patients with DM typically present with severe calcification, more complex lesions, and more comorbidities. CAC independently predicts poor PCI outcomes, including stent thrombosis and target vessel revascularization [1]. Atherectomy leads to improved procedural outcomes, yet it is utilized in less than 5% of PCI despite a high prevalence of CAC [18]. 

In this analysis, the use of OA is associated with a high procedural success rate, successful stent delivery, low rate of angiographic complications and MACCE/MACE in patients with DM and those without DM. People with DM have a higher prevalence of CAD. PCI in patients with diabetes is associated with adverse outcomes, TVR due to increased vascular inflammation, smaller vessels, platelets, and endothelial dysfunction, and death [10-12]. People with DM also have significant plaque burden and intimal hyperplasia, leading to increased in-stent restenosis [16,18]. In this study, 42% of patients with diabetes underwent OA. People with DM have a higher prevalence of CAC and aggressive forms of CAD because of increased atherogenic factors like hypertension, obesity, and dyslipidemia [19].

Consequently, CAD is more advanced and extensive in this population. In the SPIRIT IV trial, paclitaxel-eluting stents (PES) were inferior to everolimus-eluting stents (EES) in terms of 1-year target lesion failure (TLF); however, in people with diabetes, the 1-year TLF rates for EES and PES were similar (6.4 vs 6.9) [56]. This suggests that the mechanism of restenosis and response to anti-proliferative medications in people with diabetes may differ from people without diabetes. In a recent randomized trial of DES outcomes, Kedhi et al. reported that DM is an independent predictor of 1-year TVR as rates of TVR were significantly higher in people with DM [57]. 

In patients with severe CAC, PCI is complicated by a more significant risk of MACCE, including death [21]. The polymer coating of DES can be damaged during placement leading to TVR. People with severe CAC are usually excluded from randomized control trials due to their lesions' poor outcomes and complexity. Atherectomy modifies plaque leading to improved procedural success and decreased complications [22]. There is limited data on the use of atherectomy devices in people with DM. Rotational atherectomy was the most used device for treating severe CAC before the introduction of OA in 2013. OA is primarily used in Japan and the USA. People with DM had a higher restenosis rate after RA than people without DM for extensive CAD [33].

The use of OA appears to be safe in people with DM. A recent study by Tanaka et al., 2020, with the newest version of the OA device, which involved 57.4% of people with DM, resulted in a procedural success rate of 97.9% and a 2.1% 30-day incidence of MACE [58]. This is similar to the procedural success and 30-day MACE reported by our study.

The COAP-PCI prospective study (which compared the efficacy of OA and RA enrolled 51.7% of patients with DM in the OA arm) recorded a low rate of significant dissection and perforation (1.3% and 0.4%) in the OA group [59]. This is similar to the in-hospital records of this study. Another study (feasibility of OA in aortic stenosis) involving people living with diabetes recorded comparable results (2.8% 30-day MACE and 16.7% 1-year MACE) [60].

Although severe calcification is an exclusion criterion for most RCT, the 1-year TVR rate (2.8%-5.9%) observed in our study, which involved heavily calcified lesions, is lower than that of Kedhi et al. (9.4%), which is an RCT of DES outcome in people with DM [61]. Thus, the lower rates of TVR observed in our study may be explained by plaque modification by OA. Furthermore, the pivotal OA trials, which include ORBIT 1 [54], ORBIT 1 [53], and the COAST trial [62], reported improved PCI outcomes after orbital atherectomy and low rates of angiographic complications/MACE, which are comparable to the result of this study.

Strengths of This Study

Our study was reported following the 2020 PRISMA template. This study will fill an essential gap in the literature, being reviewed systematically for the first time. It may increase the number of patients with diabetes treated with PCI, an approach they prefer to CABG.

Study Limitations 

This study was conducted using mostly observational studies. The unavailability of patient-level data precluded a weighted comparison, making bias plausible. The possible confounding factors and the risk of bias (selection, reporting, and publication) may have affected the results [63]. Half of the study has a limited sample size, reducing the study's power. Most of the studies did not utilize intravascular imaging and optical coherence tomography to quantify the degree of calcification, and failed to provide detailed lesion characteristics. Thus, the observed differences could be from the lesion characteristics and not device technology. Most studies are retrospective, lack a control arm, have unequal test groups, and were not designed for people with diabetes a priori. The baseline characteristics between diverse groups may have affected the clinical outcomes. Furthermore, data were variably available, and the decision to use OA was at the operator's discretion in most cases. There were no RCTs on the efficacy of OA in patients with diabetes; however, the ongoing ECLIPSE RCT (Clinical Trials.gov identifier: NCT03108456) may help determine the superiority of OA for treating severely calcified lesions. 

## Conclusions

People with diabetes represent a significant proportion of patients with CAC. Several studies have linked CAC with unsatisfactory PCI outcomes. However, clinical outcomes following OA before PCI were similar between people living with DM and without DM. Thus, OA represents a safe and effective treatment option for CAC in people with DM. Nevertheless, multicentre randomized controlled trials are needed to confirm which atherectomy device is the most suitable for patients with DM.

Studies comparing the newer version of RA with OA are also needed to determine the ideal treatment strategy for severe CAC. Additionally, comparing the outcome of PCI with DES after OA and CABG is pertinent to verify which revascularization procedure is optimal for DM patients since atherectomy is utilized in less than 5% of patients.

## References

[1] Zhai C, Cong H, Hou K, Hu Y, Zhang J, Zhang Y (2019). Clinical outcome comparison of percutaneous coronary intervention and bypass surgery in diabetic patients with coronary artery disease: A meta-analysis of randomized controlled trials and observational studies. Diabetol Metab Syndr.

[2] Lee MS, Shah N (2016). The impact and pathophysiologic consequences of coronary artery calcium deposition in percutaneous coronary interventions. J Invasive Cardiol.

[3] Mintz GS (2015). Intravascular imaging of coronary calcification and its clinical implications. JACC Cardiovasc Imaging.

[4] Polonsky TS, McClelland RL, Jorgensen NW, Bild DE, Burke GL, Guerci AD, Greenland P (2010). Coronary artery calcium score and risk classification for coronary heart disease prediction. JAMA.

[5] Yano Y, O'Donnell CJ, Kuller L (2017). Association of coronary artery calcium score vs age with cardiovascular risk in older adults: An analysis of pooled population-based studies. JAMA Cardiol.

[6] Suvi K, Belma M, Pouya S IDF:diabetes atlas ninth edition. https://diabetesatlas.org/atlas/ninth-edition/.

[7] Setacci C, de Donato G, Setacci F, Chisci E (2009). Diabetic patients: Epidemiology and global impact. J Cardiovasc Surg (Torino).

[8] Iminger-Finger I, Kargul J, Laurent GJ (2017). Diabetes: Present and future. Int J Biochem Cell Biol.

[9] Lee MS, Shlofmitz E, Nguyen H, Shlofmitz RA (2016). Outcomes in diabetic patients undergoing orbital atherectomy system. J Interv Cardiol.

[10] Chambers JW, Behrens AN, Martinsen BJ (2016). Atherectomy devices for the treatment of calcified coronary lesions. Interv Cardiol Clin.

[11] Nicholls SJ, Tuzcu EM, Kalidindi S (2008). Effect of diabetes on progression of coronary atherosclerosis and arterial remodeling: a pooled analysis of 5 intravascular ultrasound trials. J Am Coll Cardiol.

[12] Mathew V, Gersh BJ, Williams BA (2004). Outcomes in patients with diabetes mellitus undergoing percutaneous coronary intervention in the current era: A report from the Prevention of REStenosis with Tranilast and its Outcomes (PRESTO) trial. Circulation.

[13] Lee M, Généreux P, Shlofmitz R (2017). Orbital atherectomy for treating de novo, severely calcified coronary lesions: 3-year results of the pivotal ORBIT II trial. Cardiovasc Revasc Med.

[14] Lee MS, Rha SW, Han SK (2015). Comparison of diabetic and non-diabetic patients undergoing endovascular revascularization for peripheral arterial disease. J Invasive Cardiol.

[15] Darling JD, Bodewes TC, Deery SE (2018). Outcomes after first-time lower extremity revascularization for chronic limb-threatening ischemia between patients with and without diabetes. J Vasc Surg.

[16] Lee JH, Lee SW (2013). Strategies for multivessel revascularization in patients with diabetes. J Comp Eff Res.

[17] Xin X, Wang X, Dong X, Fan Y, Shao W, Lu X, Xiao P (2019). Efficacy and safety of drug-eluting stenting compared with bypass grafting in diabetic patients with multivessel and/or left main coronary artery disease. Sci Rep.

[18] Liu X, Zhang W, Wang L, Wang S, Yu Y, Chen S, Ao H (2019). Male patients with diabetes undergoing coronary artery bypass grafting have increased major adverse cerebral and cardiovascular events. Interact Cardiovasc Thorac Surg.

[19] Bundhun PK, Wu ZJ, Chen MH (2016). Coronary artery bypass surgery compared with percutaneous coronary interventions in patients with insulin-treated type 2 diabetes mellitus: a systematic review and meta-analysis of 6 randomized controlled trials. Cardiovasc Diabetol.

[20] Bangalore S, Toklu B, Feit F (2014). Outcomes with coronary artery bypass graft surgery versus percutaneous coronary intervention for patients with diabetes mellitus: Can newer generation drug-eluting stents bridge the gap?. Circ Cardiovasc Interv.

[21] Généreux P, Madhavan MV, Mintz GS (2014). Ischemic outcomes after coronary intervention of calcified vessels in acute coronary syndromes: Pooled analysis from the HORIZONS-AMI (Harmonizing Outcomes With Revascularization and Stents in Acute Myocardial Infarction) and ACUITY (Acute Catheterization and Urgent Intervention Triage Strategy) Trials. J Am Coll Cardiol.

[22] Chambers JW, Diage T (2014). Evaluation of the diamondback 360 coronary orbital atherectomy system for treating de novo, severely calcified lesions. Expert Rev Med Devices.

[23] Leopold JA (2015). Vascular calcification: Mechanisms of vascular smooth muscle cell calcification. Trends Cardiovasc Med.

[24] Moussa I, Leon MB, Baim DS (2004). Impact of sirolimus-eluting stents on outcome in diabetic patients: A SIRIUS (SIRolImUS-coated Bx velocity balloon-expandable stent in the treatment of patients with de novo coronary artery lesions) substudy. Circulation.

[25] Moses JW, Nikolsky E, Mehran R (2006). Safety and efficacy of the 2.25-mm sirolimus-eluting Bx velocity stent in the treatment of patients with de novo native coronary artery lesions: The SIRIUS 2.25 trial. Am J Cardiol.

[26] Barbato E, Shlofmitz E, Milkas A, Shlofmitz R, Azzalini L, Colombo A (2017). State of the art: Evolving concepts in the treatment of heavily calcified and undilatable coronary stenoses - from debulking to plaque modification, a 40-year-long journey. EuroIntervention.

[27] Levine GN, Bates ER, Blankenship JC (2011). 2011 ACCF/AHA/SCAI Guideline for Percutaneous Coronary Intervention: A report of the American College of Cardiology Foundation/American Heart Association Task Force on Practice Guidelines and the Society for Cardiovascular Angiography and Interventions. Circulation.

[28] Shlofmitz E, Martinsen BJ, Lee M (2017). Orbital atherectomy for the treatment of severely calcified coronary lesions: Evidence, technique, and best practices. Expert Rev Med Devices.

[29] Bourantas CV, Zhang YJ, Garg S (2014). Prognostic implications of coronary calcification in patients with obstructive coronary artery disease treated by percutaneous coronary intervention: A patient-level pooled analysis of 7 contemporary stent trials. Heart.

[30] Arora S, Panaich SS, Patel N (2016). Coronary atherectomy in the United States (from a nationwide inpatient sample). Am J Cardiol.

[31] Mintz GS, Popma JJ, Pichard AD (1995). Patterns of calcification in coronary artery disease. A statistical analysis of intravascular ultrasound and coronary angiography in 1155 lesions. Circulation.

[32] Doshi R, Shlofmitz E, Meraj P (2017). Clinical outcomes of atherectomy prior to percutaneous coronary intervention in patients with diabetes (COAP-DM study). Catheter Cardiovasc Interv.

[33] vom Dahl J, Dietz U, Haager PK (2002). Rotational atherectomy does not reduce recurrent in-stent restenosis: Results of the angioplasty versus rotational atherectomy for treatment of diffuse in-stent restenosis trial (ARTIST). Circulation.

[34] Berry C, Tardif JC, Bourassa MG (2007). Coronary heart disease in patients with diabetes: part II: Recent advances in coronary revascularization. J Am Coll Cardiol.

[35] Chambers JW, Feldman RL, Himmelstein SI (2014). Pivotal trial to evaluate the safety and efficacy of the orbital atherectomy system in treating de novo, severely calcified coronary lesions (ORBIT II). JACC Cardiovasc Interv.

[36] Page MJ, McKenzie JE, Bossuyt PM (2021). The PRISMA 2020 statement: An updated guideline for reporting systematic reviews. BMJ.

[37] Julian PT, Higgins Higgins, James T (2019). Cochrane handbook for systematic reviews of interventions.

[38] (2023). Critical appraisal checklists. https://casp-uk.net/casp-tools-checklists/.

[39] Chambers JW, Martinsen BJ, Sturm RC (2022). Orbital atherectomy of calcified coronary ostial lesions. Catheter Cardiovasc Interv.

[40] Rao LG, Rao AM, Rao SP (2021). Outcomes after coronary orbital atherectomy at centers without on-site surgical backup: Diabetics versus non-diabetics and impact of access site. Cardiovasc Revasc Med.

[41] Beohar N, Stone GW, Martinsen BJ (2022). Coronary orbital atherectomy treatment of Hispanic and Latino patients: A real-world comparative analysis. Catheter Cardiovasc Interv.

[42] Sturm R, Martinsen BJ, Valle JA, Waldo SW, Behrens AN, Armstrong EJ (2020). Orbital atherectomy for treatment of complex severely calcified coronary artery lesions: Insights from a veterans affairs cohort. Cardiovasc Revasc Med.

[43] Shlofmitz E, Jeremias A, Goldberg A (2019). Safety of same-day discharge after percutaneous coronary intervention with orbital atherectomy. Cardiovasc Revasc Med.

[44] Lee MS, Martinsen BJ, Lee AC, Behrens AN, Shlofmitz RA, Kim CY, Chambers JW (2018). Impact of diabetes mellitus on procedural and one year clinical outcomes following treatment of severely calcified coronary lesions with the orbital atherectomy system: A subanalysis of the ORBIT II study. Catheter Cardiovasc Interv.

[45] Lee MS, Shlofmitz E, Shlofmitz R (2018). Outcomes of orbital atherectomy in severely calcified small (2.5 mm) coronary artery vessels. J Invasive Cardiol.

[46] Lee MS, Shlofmitz E, Park KW, Goldberg A, Jeremias A, Shlofmitz R (2018). Orbital atherectomy of severely calcified unprotected left main coronary artery disease: One-year outcomes. J Invasive Cardiol.

[47] Lee MS, Shlofmitz E, Goldberg A, Shlofmitz R (2018). Multicenter registry of real-world patients with severely calcified coronary lesions undergoing orbital atherectomy: 1-year outcomes. J Invasive Cardiol.

[48] Desai R, Mirza O, Martinsen BJ, Kumar G (2018). Plaque modification of severely calcified coronary lesions via orbital atherectomy: Single-center observations from a complex Veterans Affairs cohort. Health Sci Rep.

[49] Whitbeck MG, Dewar J, Behrens AN, Watkins J, Martinsen BJ (2018). Acute outcomes after coronary orbital atherectomy at a single center without on-site surgical backup: An experience in diabetics versus non-diabetics. Cardiovasc Revasc Med.

[50] Lee MS, Shlofmitz E, Lluri G, Shlofmitz R (2017). Impact of impaired renal function in patients with severely calcified coronary lesions treated with orbital atherectomy. J Invasive Cardiol.

[51] Généreux P, Bettinger N, Redfors B (2016). Two-year outcomes after treatment of severely calcified coronary lesions with the orbital atherectomy system and the impact of stent types: Insight from the ORBIT II trial. Catheter Cardiovasc Interv.

[52] Bhatt P, Parikh P, Patel A, Chag M, Chandarana A, Parikh R, Parikh K (2015). Long-term safety and performance of the orbital atherectomy system for treating calcified coronary artery lesions: 5-Year follow-up in the ORBIT I trial. Cardiovasc Revasc Med.

[53] Bhatt P, Parikh P, Patel A, Chag M, Chandarana A, Parikh R, Parikh K (2014). Orbital atherectomy system in treating calcified coronary lesions: 3-Year follow-up in first human use study (ORBIT I trial). Cardiovasc Revasc Med.

[54] Parikh K, Chandra P, Choksi N, Khanna P, Chambers J (2013). Safety and feasibility of orbital atherectomy for the treatment of calcified coronary lesions: The ORBIT I trial. Catheter Cardiovasc Interv.

[55] Boon MH, Thomson H (2021). The effect direction plot revisited: Application of the 2019 Cochrane Handbook guidance on alternative synthesis methods. Res Synth Methods.

[56] Stone GW, Rizvi A, Newman W (2010). Everolimus-eluting versus paclitaxel-eluting stents in coronary artery disease. N Engl J Med.

[57] Hermanides RS, Kennedy MW, Kedhi E (2020). Impact of elevated HbA1c on long-term mortality in patients presenting with acute myocardial infarction in daily clinical practice: Insights from a 'real world' prospective registry of the Zwolle Myocardial Infarction Study Group. Eur Heart J Acute Cardiovasc Care.

[58] Tanaka Y, Yokota S, Hayashi T (2020). Procedural and clinical outcomes with the diamondback 360® orbital atherectomy system: A classic crown with glideassist® and viperwire advance® flextip. J Am Coll Cardiol.

[59] Meraj PM, Shlofmitz E, Kaplan B, Jauhar R, Doshi R (2018). Clinical outcomes of atherectomy prior to percutaneous coronary intervention: A comparison of outcomes following rotational versus orbital atherectomy (COAP-PCI study). J Interv Cardiol.

[60] Shlofmitz E, Goldberg AH, Pollack S (2019). Safety and feasibility of orbital atherectomy in patients with aortic stenosis. JACC Cardiovasc Interv.

[61] Kedhi E, Généreux P, Palmerini T (2014). Impact of coronary lesion complexity on drug-eluting stent outcomes in patients with and without diabetes mellitus: Analysis from 18 pooled randomized trials. J Am Coll Cardiol.

[62] Redfors B, Sharma SK, Saito S (2020). Novel micro crown orbital atherectomy for severe lesion calcification: Coronary Orbital Atherectomy System Study (COAST). Circ Cardiovasc Interv.

[63] Hess AS, Abd-Elsayed A (2019). Observational studies: uses and limitations. Pain.

